# Caring for Caregivers of Older Adults: A Quality Improvement Innovation Approach

**DOI:** 10.1093/geroni/igaf122.3893

**Published:** 2025-12-31

**Authors:** Diane Mariani, Alison Wagner

**Affiliations:** Rush University Medical Center, Chicago, Illinois, United States; Rush University Medical Center, Chicago, Illinois, United States

## Abstract

Caregivers of older adults in the United States are crucial partners in the health and well-being of older adult care recipients. Despite their key role in caring for the nation’s older adults, which is in existence due to the nation’s lack of a plan for care for those who are not in need of residential care, caregivers are rarely asked whether or not they are ready, willing or even able to provide care, and neither are their needs identified or addressed within health systems. Caring for Caregivers (C4C) was developed at Rush University Medical Center to create system change by addressing how caregivers are identified within an Age-Friendly Health Systems, and to provide services and support for family caregivers that focus on their physical and emotional health and well-being. The first model to formally integrate caregivers into Age-Friendly Health Systems, C4C provides a comprehensive assessment of needs, strengths, and the 4Ms namely What Matters, Medication, Mentation, Mentation, and Mobility and then develops an individualized intervention plan for each caregiver. Follow up assessments for burden depression, and anxiety are completed at both one-month and three-month post intervention. Late-breaking data demonstrating statistically significant reductions in burden, depression, and anxiety symptoms for caregivers will be shared. Caregivers who completed an initial assessment and at least one follow-up assessment after completing the intervention, demonstrated a decrease in symptoms of depression via PHQ-9 (n = 47; z= -134.5, p = 0.03), anxiety via GAD-7 (n = 46; z = 203.5, p = 0.002), and caregiver burden via the Burden Scale for Caregivers (n = 45; z=-193.5, p = 0.01).

